# Supplementary material for: A HIMU-like component in Mariana Convergent Margin magma sources during initial arc rifting revealed by melt inclusions
Source: Nat Commun. 2024 May 14;15:4088. doi: 10.1038/s41467-024-48308-y (PMC11094193; doi:10.1038/s41467-024-48308-y)
Supplement: Supplementary file 1 — Supplementary Information [file 41467_2024_48308_MOESM1_ESM.pdf]

Supplementary Information

A HIMU-like component in Mariana Convergent Margin magma sources during initial arc rifting revealed by melt inclusions

Xiaohui Li[1,2,3], Osamu Ishizuka[4,5], Robert J. Stern[6], Sanzhong Li[1,2*], Zhiqing Lai[1], Ian Somerville[7], Yanhui Suo[1,2], Long Chen[1,2], Hongxia Yu[8]

This file includes:

- Major and trace element analysis
- Supplementary Figures 1-7
- Supplementary References

1. Major and trace element measurements

Major element compositions were determined using an X-ray fluorescence (XRF) spectrometer at the Institute of Oceanology, Chinese Academy of Sciences (IOCAS). The sample powders (~0.6 g) were mixed with 6.0 g of lithium tetraborate ($Li_2B_4O_7$) to make glass disks in an automatic melting instrument at 1050–1100 °C. The loss on ignition (LOI) values were calculated based on the weight differences after ignition at 1000 °C. The analytical accuracy was assessed using certified reference materials BHVO-2, BCR-2, AGV-2, and BIR-1 and the measured values of the major elements are within the recommended ranges (Supplementary Table 2). Trace element analyses were conducted using inductively coupled plasma-mass spectrometry (ICP-MS) (ICAP TQ) at IOCAS. During the trace element analysis, 50 mg powder samples were accurately weighed and placed in Teflon beakers. Then, 1.0 ml concentrated HF was added to the beakers, and the beaker was closed and heated at 50 °C for 24 h. After this, the beakers were opened and heated at 130 °C so that the solution evaporated until incipient dryness. Then, 1.5 ml concentrated HF and 1.0 ml HNO_3 were added, and transferred the sample solutions to the digestion bomb units, which consisted of a stainless-steel bomb jacket and a Teflon capsule. The bomb units were heated in an oven at 190 °C for 72 h. Afterward, cooled the bomb units and removed the Teflon capsules from the steel jacket, opened and heated them on a hotplate at 160 °C to evaporate the solutions until dryness. Then, redissolved the residues with concentrated HNO_3, and sealed and heated in the Teflon capsules on a hotplates at 130 °C for 30 min.

The solutions were diluted 1000 times with 1% distilled HNO_3 and then analyzed by ICP-MS. BHVO-2 and BCR-2 were used as external standards to evaluate their analytical accuracy, and the results are shown in Supplementary Table 2. The analytical accuracy of most measured trace elements was better than 5%.

2. Supplementary Figures

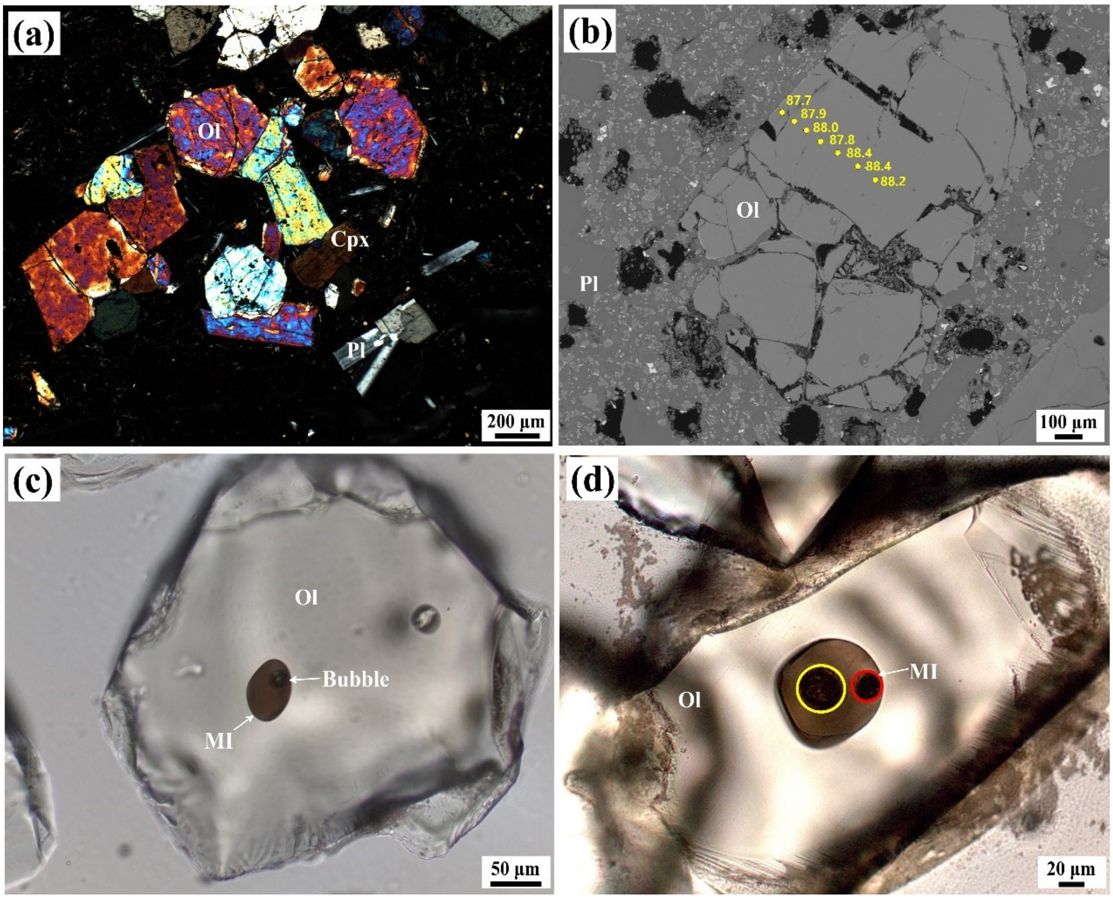

Supplementary Fig. 1. Representative photomicrographs of minerals (a), back-scattered electron images (BSE) of olivine (b), and optical images of melt inclusions (c, d) in basalts form NSP-24. MI - melt inclusion; Ol - olivine; Cpx - clinopyroxene; Pl - plagioclase. Red circle in (d) denotes LA-ICP-MS analytical spots for trace elements and yellow circle denotes the LA-MC-ICP-MS analytical spots for Pb isotopes.

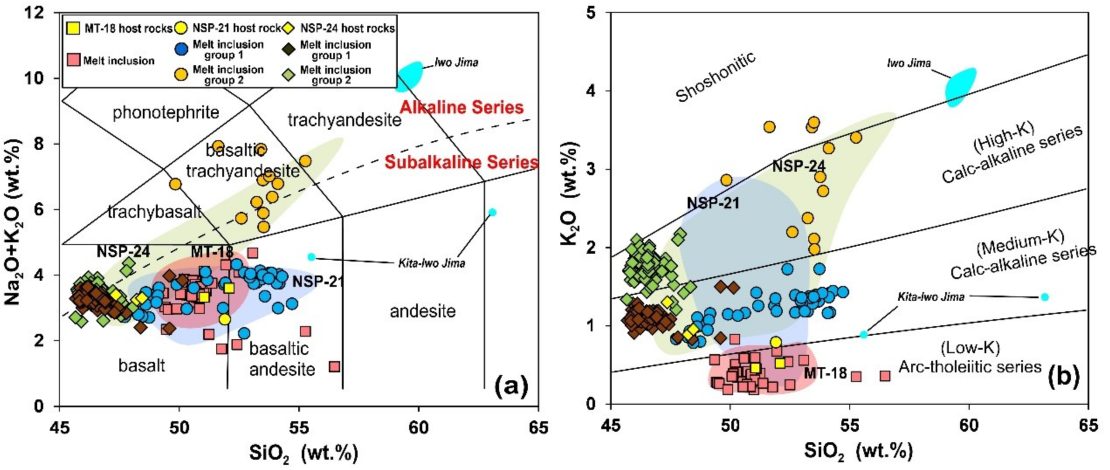

Supplementary Fig. 2. Plots of corrected melt inclusion and whole-rock compositions on the total alkali-silica classification diagram (Na_2O+K_2O versus SiO_2)[1] (a), and SiO_2 versus K_2O diagram[2] (b). The fields of whole-rocks from NSP-24[3,4,5], NSP-21[5,6], MT-18[6,7,8], Iwo Jima[4], and Kita-Iwo Jima[9] are also shown for comparison.

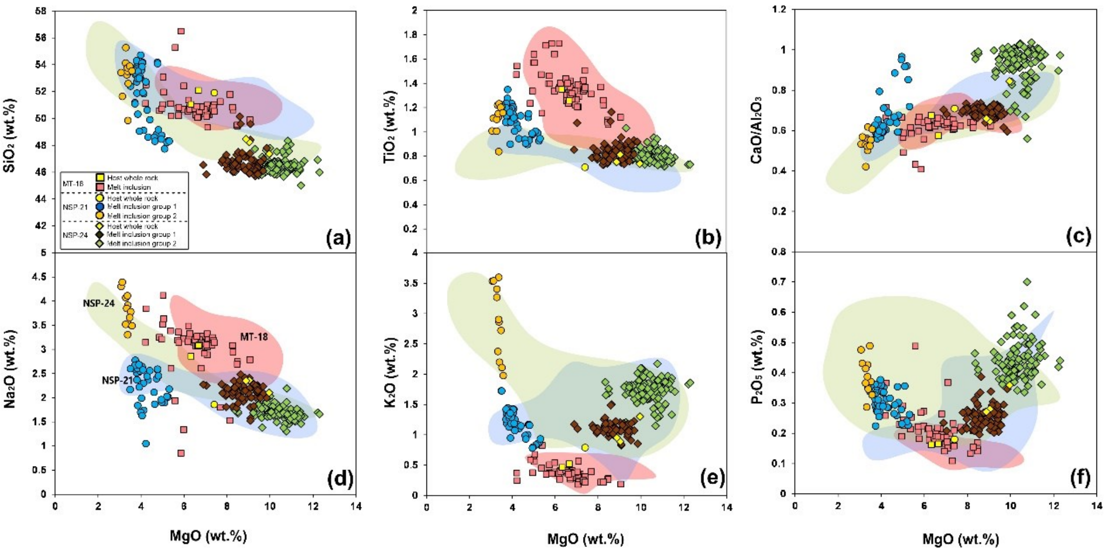

Supplementary Fig. 3. Variations in SiO_2 (a), TiO_2 (b), CaO/Al_2O_3 (c), Na_2O (d), K_2O (e), and P_2O_5 (f) versus MgO in the corrected melt inclusions and their host whole-rocks measured in this study. The fields of studied whole-rocks from NSP-24, NSP-21, and MT-18 are the same as in Supplementary Fig. 1.

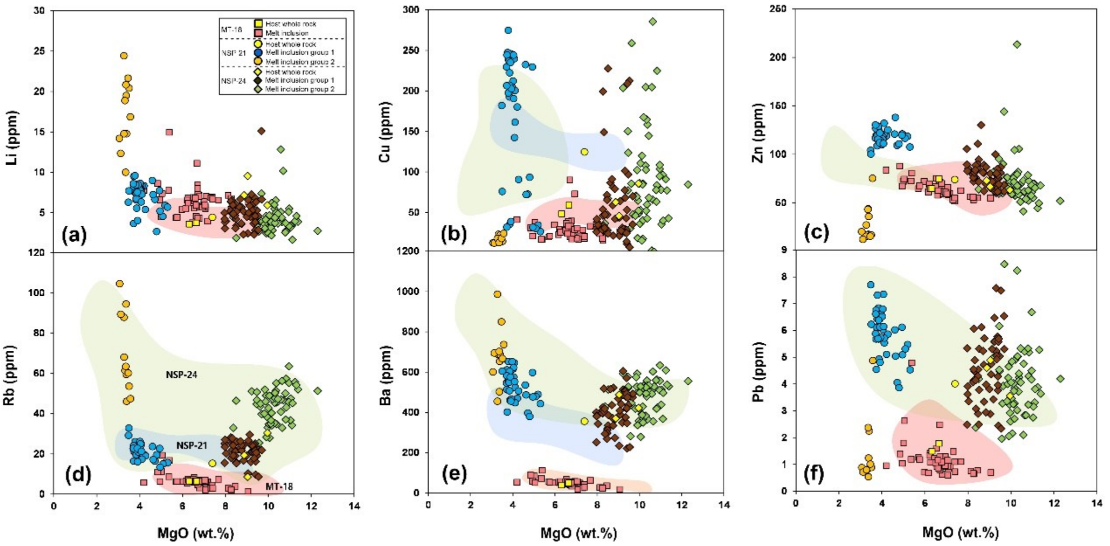

Supplementary Fig. 4. MgO plotted against trace elements of Li (a), Cu (b), Zn (c), Rb (d), Ba (e), and Pb (f) in the corrected melt inclusions and their hosted whole-rocks. The fields of studied whole-rocks from NSP-24, NSP-21, and MT-18 are the same as in Supplementary Fig. 1.

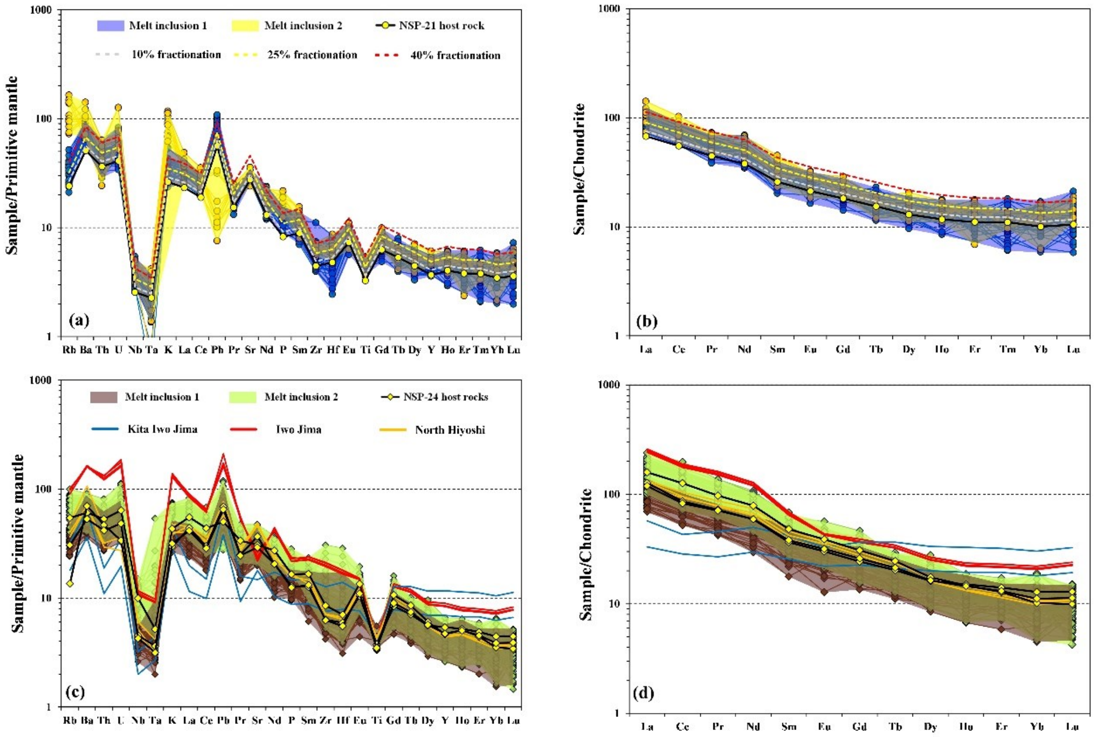

Supplementary Fig. 5. Simulated calculations of trace and rare earth element compositions of NSP-21 melt inclusions (a and b). The compositional variations of the group 1 melt inclusions result from < 40% fractional crystallization of melt in equilibrium with NSP-21 host rock that remove only olivine. Trace and rare earth element compositions of NSP-24 melt inclusions and the lavas in the adjacent area (c and d). The compositions of trace and rare earth elements in NSP-24 melt inclusions and their host whole rock are similar to that of AVP basaltic lavas (e.g., North Hiyosh, 23°22′N) but different from the andesitic-dacitic seamount volcanic rocks from Iwo Jima (24°45′N) and Kita Iwo Jima (25°30′N) in the southern Izu-Bonin arc.

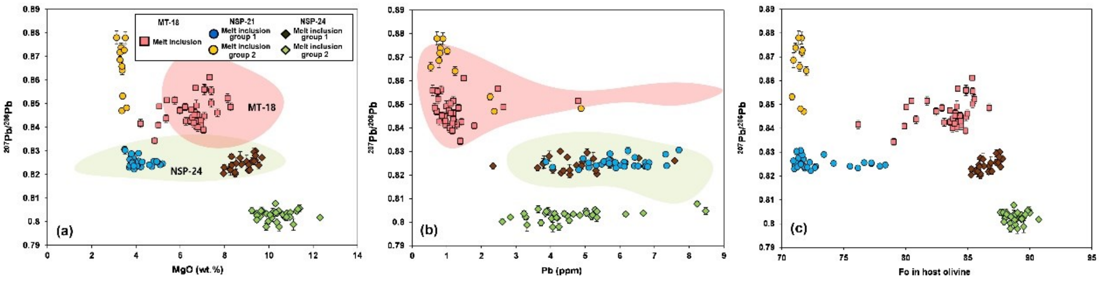

Supplementary Fig. 6. Variations of MgO (a), Pb (b), and olivine Fo (c) versus $^{207}Pb/^{206}Pb$ in the corrected melt inclusions. Error bars with the $^{207}Pb/^{206}Pb$ data of this study are 2 standard errors (2SE). The fields of studied whole-rocks from NSP-24 and MT-18 are the same as in Supplementary Fig. 1.

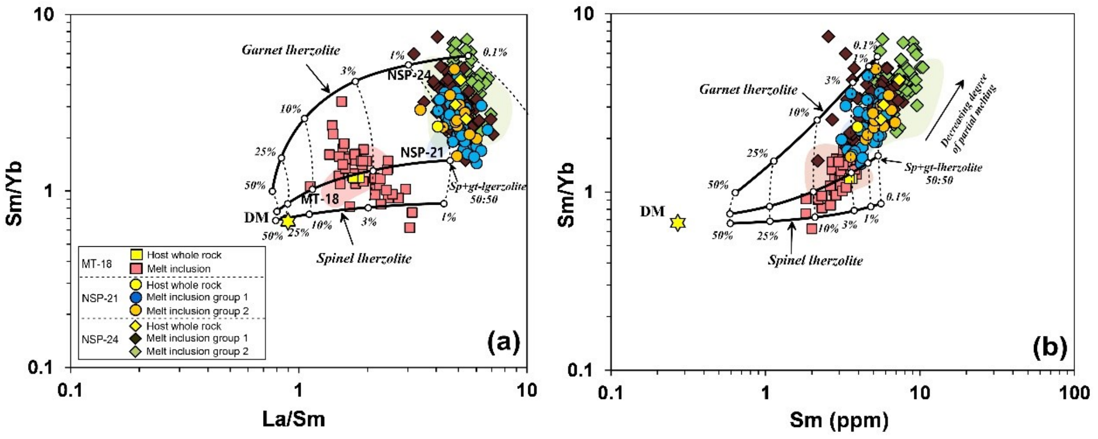

Supplementary Fig. 7. Plot of Sm/Yb versus La/Sm (a) and Sm/Yb versus Sm (b) for the studied melt inclusions, showing that the source region compositions and degree of partial melting varies for different regions. Depleted mantle (DM) with La = 0.234, Sm = 0.270, and Yb = 0.401[10] was modeled with varying degrees of batch melting (F) and mineral compositions. The mode of garnet lherzolite was taken as $Ol_{0.600}$ + $Opx_{0.200}$ + $Cpx_{0.100}$ + $Gt_{0.100}$ and that of the spinel lherzolite as $Ol_{0.530}$ + $Opx_{0.270}$ + $Cpx_{0.170}$ + $Sp_{0.030}$. Phase proportions entering the melt were taken as $Ol_{0.030}$ + $Opx_{0.160}$ + $Cpx_{0.880}$ + $Gt_{0.090}$ and $Ol_{0.060}$ + $Opx_{0.280}$ + $Cpx_{0.670}$ + $Sp_{0.110}$, respectively[11]. The partition coefficients (D) of minerals in melt are from ref. 12. (Ol-olivine; Cpx-clinopyroxene; Opx-orthopyroxene; Sp-spinel; and Gt-garnet).

3. Supplementary References

1. Middlemost, E. A. Naming materials in the magma/igneous rock system. *Earth-sci Rev*. **37**, 215-224 (1994).
2. Peccerillo, A. & Taylor, S.R. Geochemistry of the Eocene calc-alkaline volcanic rocks in the Kastamonu area, northern Turkey. *Contrib. Miner. Petrol.* **58**, 63-81

(1976).

3. Ishizuka, O. et al. Migrating shoshonitic magmatism tracks Izu-Bonin-Mariana intra-oceanic arc rift propagation. *Earth Planet. Sci. Lett*. **294**, 111-122 (2010).
4. Sun, C. H. & Stern, R. J. Genesis of Mariana shoshonites: Contribution of the subduction component. *J. Geophys. Res. Solid Earth* **106**, 589-608 (2001).
5. Bloomer, S.H., Stern, R.J., Fisk, E. & Geschwind, C.H. Shoshonitic volcanism in the northern Mariana Arc: 1. petrographic and major and trace element characteristics. *J. Geophys. Res.* **94**, 4469–4496 (1989).
6. Li, X. et al. Across-arc variations in Mo isotopes and implications for subducted oceanic crust in the source of back-arc basin volcanic rocks. *Geology* **49**, 1165-1170 (2021).
7. Zhao, G., Luo, W., Lai, Z., Tian, L. & Xu, C. Influence of subduction components on magma composition in back-arc basins: a comparison between the Mariana and Okinawa troughs. *Geol. J.* **51**, 357-367 (2016).
8. Yan, Q. et al. Geochemistry of axial lavas from the mid-and southern Mariana Trough, and implications for back-arc magmatic processes. *Mineral. Petrol.* **113**, 803-820 (2019).
9. Ishizuka, O., Taylor, R. N., Yuasa, M., Milton, J. A., Nesbitt, R. W., Uto, K., & Sakamoto, I. Processes controlling along ‐ arc isotopic variation of the southern Izu ‐ Bonin arc. *Geochem. Geophys. Geosyst*. **8(6)** (2007).
10. Salters, V. J. & Stracke, A. Composition of the depleted mantle. *Geochem. Geophys.*

Geosyst. **5**, Q05004 (2004).

11. Aldanmaz, E., Pearce, J. A., Thirlwall, M. F., & Mitchell, J. G. Petrogenetic evolution of late Cenozoic, post-collision volcanism in western Anatolia, Turkey. *J. Volcanol. Geotherm. Res*. **102(1-2)**, 67-95. (2000).
12. McKenzie, D.P. & O'Nions, R.K. Partial melt distribution from inversion of rare earth element concentrations. *J. Petrol*. **32**, 1021-1991 (1991).
