# Supplementary material for: A HIMU-like component in Mariana Convergent Margin magma sources during initial arc rifting revealed by melt inclusions
Source: Nat Commun. 2024 May 14;15:4088. doi: 10.1038/s41467-024-48308-y (PMC11094193; doi:10.1038/s41467-024-48308-y)
Supplement: Supplementary file 3 — Description of Additional Supplementary Files [file 41467_2024_48308_MOESM3_ESM.pdf]

Description of Additional Supplementary Files

Supplementary Data 1: Brief description of studied host volcanic rocks.

Supplementary Data 2: Major, trace element and Pb isotope compositions of the studied host whole -rock samples and standard samples.

Supplementary Data 3: Compositions of melt inclusions and their host olivines.

Supplementary Data 4: The measured and recommended trace element compositions of the reference glass analysis by LA-ICP-MS.

Supplementary Data 5: Analytical Pb isotopic ratios of standard samples.

Supplementary Data 6: Parameters for the mixing calculations in Fig. 3.
